# Antihyperglycemic drugs use and new-onset atrial fibrillation: A population-based nested case control study

**DOI:** 10.1371/journal.pone.0197245

**Published:** 2018-08-30

**Authors:** Yi-Sheng Liou, Fu-Yu Yang, Hung-Yi Chen, Gwo-Ping Jong

**Affiliations:** 1 Department of Family Medicine and Geriatrics, Taichung Veteran General Hospital, and School of Public Health, National Defense Medical Center, Taipei, Taiwan, ROC; 2 Institute of Pharmacy, China Medical University, Taichung, Taiwan, ROC; 3 Department of Pharmacy, China Medical University Beigang Hospital, Yunlin County, Taiwan, ROC; 4 Division of Internal Cardiology, Chung Shan Medical University Hospital and Chung Shan Medical University, Taichung, Taiwan, ROC; Osaka University Graduate School of Medicine, JAPAN

## Abstract

Currently, the potential risk of atrial fibrillation associated with antihyperglycemic drug use has been a topic of considerable interest. However, it remains uncertain whether different classes of antihyperglycemic drug therapy are associated with the risk of atrial fibrillation risk. Here, we investigated the association between different classes of antihyperglycemic drugs and new-onset atrial fibrillation (NAF). A case-matched study was performed based on the National Health Insurance Program in Taiwan. Patients who had NAF were considered the NAF group and were matched in a 1:4 ratio with patients without NAF, who were assigned to the non-NAF group. Patients were matched according to sex, age, diabetes mellitus duration, index date, and Charlson Comorbidity Index score. We used multivariate logistic regression controlling for potential confounders to examine the association between different classes of antihyperglycemic drug use and the risk of NAF. Overall, we identified 2,882 cases and 11,528 matched controls for the study. After adjusting for sex, age, comorbidities, and concurrent medications, users of biguanides or thiazolidinediones were at a lower risk of developing NAF when compared with non-users (odds ratio [OR] 0.81, 95% confidence interval [CI] 0.71–0.95 and OR 0.72, 95% CI 0.63–0.83, respectively). In contrast, users of insulin were at a higher risk of developing NAF than were non-users (OR 1.19, 95% CI 1.06–1.35). Sulfonylureas, glinides, α-glucosidase inhibitors, and dipeptidyl peptidase-4 inhibitors were not associated with developing the risk of NAF. In conclusion, the use of biguanides or thiazolidinediones may be associated with a low risk of NAF, whereas insulin may be associated with a significant increase in the risk of NAF in patients with type 2 diabetes mellitus during long-term follow-up. Further prospective randomized studies should investigate which specific class of antihyperglycemic drug treatment for diabetes mellitus can prevent or postpone NAF.

## Introduction

Diabetes mellitus (DM) has been a major public health and medical concern in both developed and developing countries [1,2]. Atrial fibrillation is a common arrhythmia [3] that has been associated with an increased risk of all-cause mortality in population-based studies [4–6]. Concerns about DM and comorbidity with atrial fibrillation, particularly new-onset atrial fibrillation (NAF) are gradually increasing worldwide [7,8]. Some recent studies on DM led to a debate about whether the use of antihyperglycemic drugs is associated with NAF in treated DM patients [9–12]. It seems obvious that the cardiovascular risk is higher when DM and atrial fibrillation coexist than when either of the two conditions stands alone; however, current reports on the effect of various antihyperglycemic drugs on the risk of NAF are conflicting. Data from these studies comparing large groups of patients who receive more than two classes of drugs are lacking [12]. Notably, compared with other antihyperglycemic drug classes used in patients with DM, it is not completely clear whether certain antihyperglycemic drug classes are associated with a higher risk of NAF. Therefore, we conducted a nested case control study to explore the relationship between all antihyperglycemic drugs and NAF in a general population in Taiwan. We aimed to determine whether insulin, biguanides, sulfonylureas, glinides, α-glucosidase inhibitors, thiazolidinediones (TZDs), and dipeptidyl peptidase-4 (DPP4) inhibitors were independently associated with NAF.

## Materials and methods

### Patient selection

This study extracted data from the National Health Insurance (NHI) program in Taiwan from January 2002 to December 2013. The Taiwan NHI program has provided comprehensive health insurance coverage for all Taiwan residents since 1995. Currently, 99% of more than 23 million enrollees are covered under the NHI program [13]. The NHI database stores information from the claim forms in two tables: one for visits and one for prescriptions. The table on visits contains patient identification numbers, sex, age, three diagnostic codes, and medical expenditures, as well as hospital and physician information. The table on prescriptions contains the quantity and expenditure for all drugs, drug dose, operations, and treatments. The details of the program have been well documented in previously published articles [13,14].

We used the International Classification of Diseases, Ninth Revision, Clinical Modification (ICD-9-CM) code to define atrial fibrillation (code 427.31) and DM (code 250). Prescriptions for antihyperglycemic drugs in patients with newly diagnosed DM before the index date were retrieved from a prescription drug database between January 2004 and December 2013. The newly diagnosed DM was defined as the first time that a type 2 DM code appeared in the inpatient or outpatient claim records between January 2004 and December 2013. DM patients with an atrial fibrillation diagnosis during the 2-year period prior to January 1, 2004 were excluded. This study was approved by the ethics committee of the Chung Shan Medical University Hospital (CSMUH-2016-REC2-16). Informed consent was waived owing to the retrospective nature of the study.

### Study design

**Fig 1:**
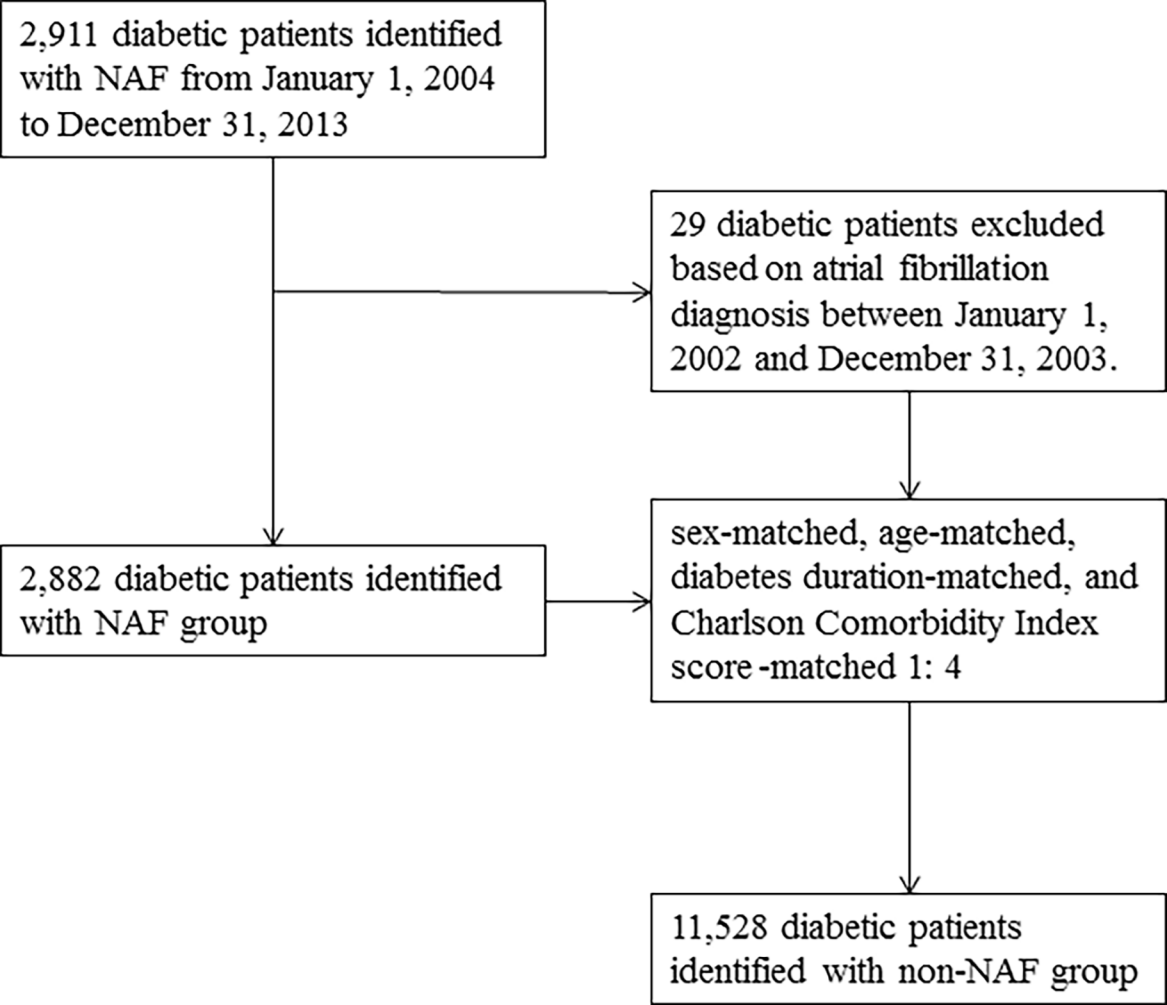
Flowchart of selection of patients for the inclusion in this study.

Finally, subjects were stratified at a 1:4 ratio into a study group comprising 2,882 participants aged 20 years and older who were newly diagnosed with atrial fibrillation from 2004 to 2013, and 11,528 sex-matched, age-matched, DM duration-matched, index date-matched, and Charlson Comorbidity Index score-matched [15] randomly selected participants without NAF served as control group (Fig 1). The index date was defined as the development of NAF, which was defined as the first time that an atrial fibrillation code appeared in the outpatient claim records. We identified all prescriptions for antihyperglycemic drugs administered to patients with NAF within a 10-year period before the date on which NAF was diagnosed. In Taiwan, all antihyperglycemic drugs were available only by prescription during the time period studied. Patients who had used one class of antihyperglycemic drug continuously before 6 months prior to the date of NAF diagnosis were categorized according to the antihyperglycemic drug class that they took: insulin, biguanides, sulfonylureas, glinides, α-glucosidase inhibitors, TZDs, or DPP4 inhibitors.

### Statistics

Continuous variables in this study are presented as mean ± standard deviation and compared using the Student’s t test. Categorical variables are presented as number and percentage, and compared by the Chi-Square test. This study was designed to reveal whether antihyperglycemic drug classes of diabetic patients are association with incident of NAF. Logistic regression analyses were applied to examine if the odds ratio (OR) for user versus non-user of antihyperglycemic agents of the NAF group is differ from that of the Non-NAF group. Additional adjusted multivariate logistic regression models including sex, age, comorbidity, and concurrent medication were implemented. All statistical tests were conducted using the SAS 9.3 (SAS Inc., Chicago, North Carolina, USA). A *p*-value of <0.05 was considered statistically significant.

## Results

### Baseline characteristics of patients

The baseline characteristics of the study population are shown in Table 1. Sex, age, and DM duration were similar between the two groups of patients, following Charlson Comorbidity Index score matching. Men comprised more than half of the sample population (7,554, 52.4%). Approximately 19.0% (2,739) of the patients took insulin, 53.8% (7,756) took biguanides, 51.7% (7,443) took sulfonylureas, 17.1% (2,459) took glinides, 23.0% (3,308) took α-glucosidase inhibitors, 20.9% (3,009) took thiazolidinediones, and 18.0% (2,595) took DPP4 inhibitors. No significant differences were observed regarding the comorbidities of obstructive sleep apnea (*p* = 1.00), hyperthyroidism (*p* = 0.81), and peripheral artery occlusive disease (*p* = 0.51) between the two groups of subjects. However, patients in the NAF group had hypertension in significantly higher numbers, as well as congestive heart failure, chronic kidney disease, acute myocardial infarction, and ischemic stroke (*p* < 0.0001). In addition, significant differences were observed regarding the concurrent therapies of antihypertensive drugs, statins, and steroids between the two groups of patients (*p* < 0.0001).

**Table 1:** Baseline characteristics of all patients.

Variable	Non-AF group N = 11528	AF group N = 2882	p-value
Age, year (SD)	69.0	69.0	0.84
DM duration, year (SD)	3.93	3.93	0.96
Sex			0.56
Female (%)	5499(47.7)	1357(47.1)	
Male (%)	6029(52.3)	1525(52.9)	
antihyperglycemia			
Insulin (%)	2009 (17.4)	730 (25.3)	<0.0001
Biguanide (%)	6162 (53.5)	1594 (55.3)	0.0758
Sulfonylurea (%)	5866 (50.9)	1577 (54.7)	0.0002
Glinide (%)	1830 (15.9)	629 (21.8)	<0.0001
α-glucosidase inhibitor (%)	2568 (22.3)	740 (25.7)	0.0001
Thiazolidinedione (%)	2385 (20.7)	624 (21.7)	0.2597
DPP-4 inhibitor (%)	2025 (17.6)	570 (19.8)	0.0062
Comorbidities			
Hypertension (%)	2119 (18.4)	704 (24.4)	<0.0001
CHF (%)	46 (0.40)	47 (1.63)	<0.0001
CKD (%)	136 (1.18)	61 (2.12)	0.0002
AMI (%)	6(0.05)	6 (0.21)	0.0194
OSA (%)	1 (0.01)	0(0.0)	1.0000
Hyperthyroidism (%)	22 (0.19)	4 (0.14)	0.8057
Ischemic stroke (%)	155 (1.34)	63 (2.2)	0.0015
PAOD (%)	24 (0.21)	8 (0.28)	0.5057
Concurrent medication			
ACEI (%)	6079(52.7)	2014 (69.9)	< .0001
ARB (%)	6189 (53.7)	2053 (71.2)	< .0001
Thiazide (%)	3436 (29.8)	1370 (47.54)	< .0001
Diuretics (%)	4825 (41.9)	2075 (72.0)	< .0001
α-blocker (%)	4666 (40.5)	1504 (52.2)	< .0001
β-blocker (%)	7933 (68.2)	2549 (88.5)	< .0001
DHP CCB (%)	8016 (69.5)	2349 (81.5)	< .0001
Non-DHP CCB(%)	3248 (28.2)	1695 (58.8)	< .0001
Statin (%)	6210 (52.9)	1615 (56.0)	0.0366
Steroid (%)	11345 (98.4)	2861 (99.3)	0.0003

SD: Standard deviation; DM: Diabetes Mellitus; DPP4: Dipeptidyl peptidase 4; CHF: Congestive heart failure; CKD: Chronic kidney disease; AMI: Acute myocardial infarction; OSA: Obstructive sleep apnea; PAOD: Peripheral artery occlusive disease; ACEI: Angiotensin converting enzyme inhibitor. ARB: Angiotensin receptor blocker. DHP: Dihydropyridine; CCB: Calcium channel blocker.

### The crude risk estimate of NAF

**Fig 2:**
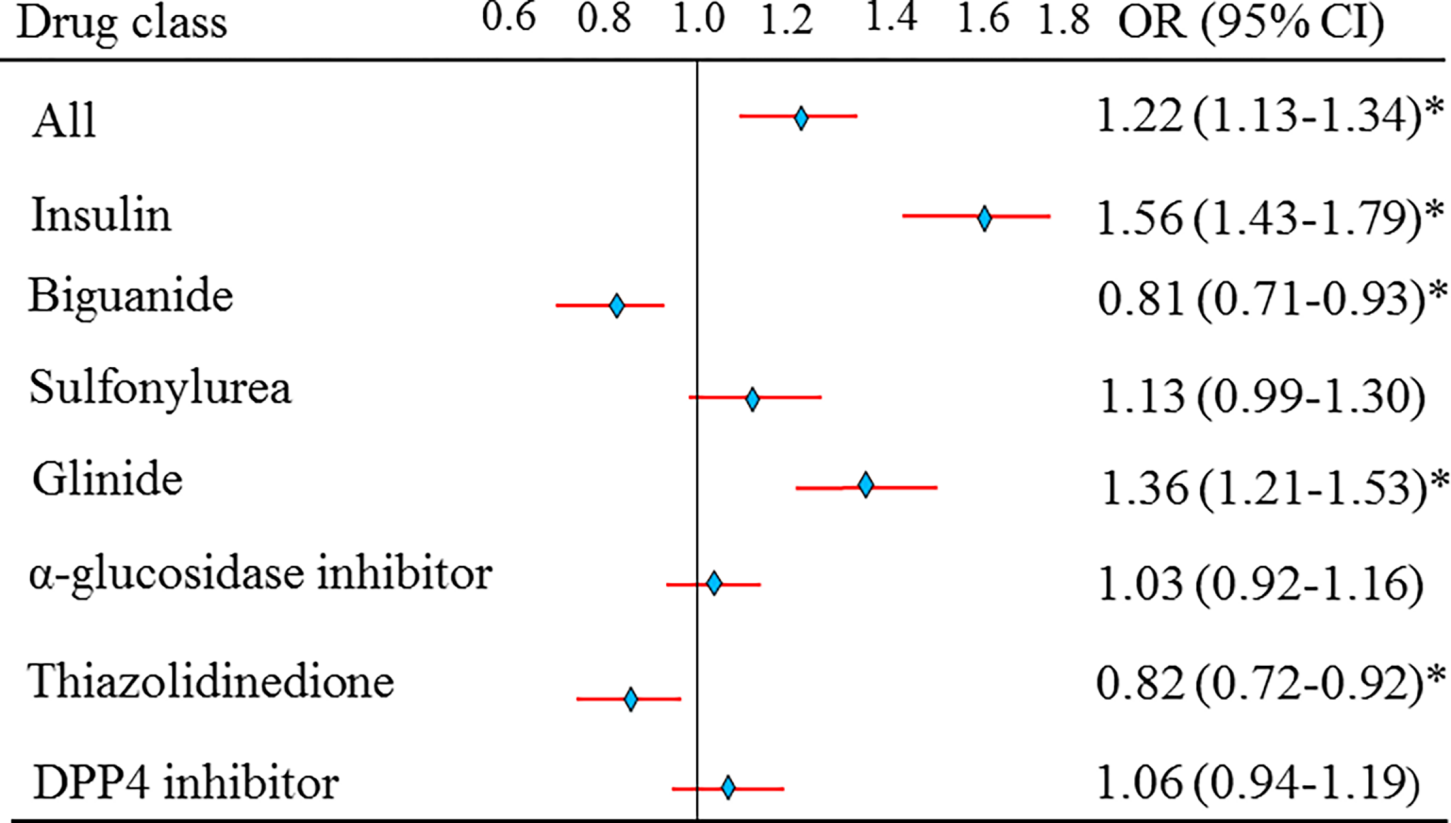
Risk of antihyperglycemic drugs on the ORs for new-onset atrial fibrillation between two groups of patients. A: Crude estimates of all antihyperglycemic drugs. *p value < 0.05.

The overall risk of NAF was significantly different [odds ratio (OR) 1.22, 95% confidence interval (CI) 1.13–1.34, *p* < 0.05] between the two groups of subjects (Fig 2). The crude risk estimate of NAF for users of biguanide (OR 0.81, 95% CI 0.71–0.93) and thiazolidinedione (OR 0.82, 95% CI 0.72–0.92) was lower than that for non-users (*p* < 0.05). Sulfonylurea (OR 1.13, 95% CI 0.99–1.30), α-glucosidase inhibitor (OR 1.03, 95% CI 0.92–1.16), and DPP4 inhibitors (OR 1.06, 95% CI 0.94–1.19) were not associated with an increased risk of NAF (*p* > 0.05). However, insulin (OR 1.56, 95% CI 1.56–1.79) and glinides (OR 1.36, 95% CI 1.21–1.53) had the highest risk estimates of NAF (*p* < 0.05) (Fig 2).

### The risk estimate of NAF after adjusting for sex, age, diabetes duration, comorbidities, and concurrent medication

**Fig 3:**
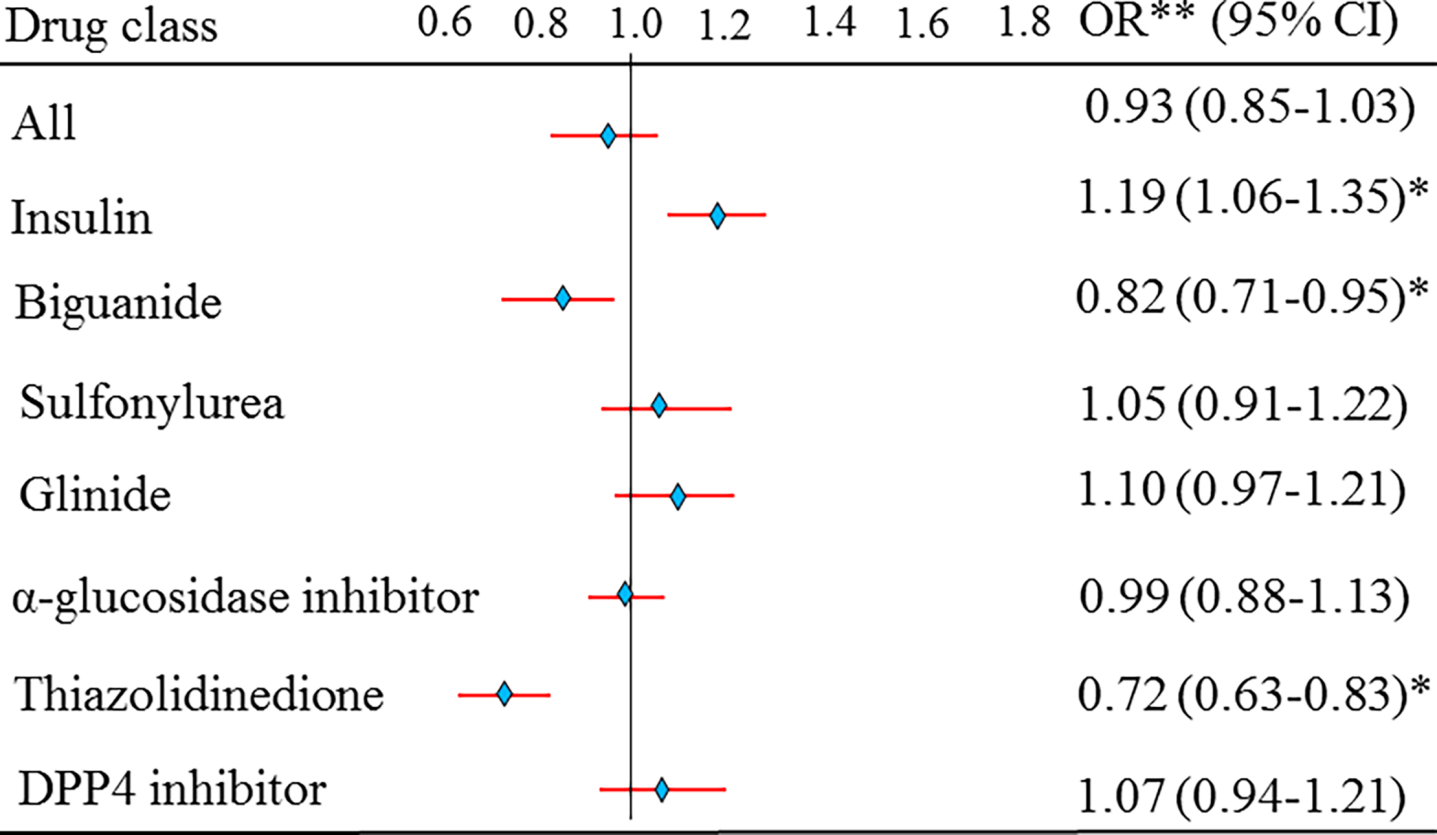
Adjusted for age, sex, comorbidities, and concurrent medication estimates of all antihyperglycemic drugs. *p value < 0.05.

Finally, the overall risk of NAF was not different (OR, 0.93; 95% CI, 0.85–1.03, *p* > 0.05) after adjusting for sex, age, DM duration, comorbidities, and concurrent medication between the two groups of subjects (Fig 3). The risk estimate of NAF after adjusting for sex, age, DM duration, comorbidities, and concurrent medication for users of insulin (OR 1.19, 95% CI 1.06–1.35) was higher (*p* < 0.05) than that for non-users. Sulfonylurea (OR 1.05, 95% CI 0.91–1.22), glinides (OR 1.10, 95% CI 0.97–1.21), α-glucosidase inhibitors (OR 0.99, 95% CI 0.88–1.13), and DPP4 inhibitors (OR 1.07, 95% CI 0.94–1.21) were not associated with an increased risk of NAF (*p* > 0.05). However, biguanide (OR 0.82, 95% CI 0.71–0.95) and TZD (OR 0.72, 95% CI 0.63–0.83) had a lower risk estimate of NAF (*p* < 0.05) (Fig 3).

## Discussion

In this nested case control study, based on a health insurance claims database, we clearly demonstrated that the use of biguanides and TZDs were associated with a lower risk of NAF, while sulfonylureas, glinides, α-glucosidase inhibitors, and DPP4 inhibitors were not associated with an increase in the risk of NAF. Insulin was associated with a significant increase in the risk of NAF. Our findings may provide important clinical implications for the mechanistic links between antihyperglycemic drugs and NAF in DM patients.

Some studies have reported that atrial fibrillation is an inflammatory process involving arrhythmia, and it increases oxidative stress and induces structural remodeling in atrial myocytes [16–18]. Under this mechanism, previous studies have also reported that metformin is associated with a lower risk of NAF development, which would be attenuated by evidence of reducing blood pressure, an anti-inflammatory response and oxidative stress in DM patients [12,19–22]. In accordance with previous findings, our results do support the association between biguanides and NAF given that NAF patients had lower odds of exposure to biguanides.

In this study, TZDs were also found to be associated with a lower risk of NAF in DM patients. Previous animal studies have clearly demonstrated that TZDs may decrease NAF risk by several mechanisms [10,22–26]. Thiazolidinediones may have a positive effect on anti-inflammatory and antioxidant activities by reducing atrial fibrosis, inflammatory response, and oxidative stress; suppressing atrial fibrillation inducibility; and reducing atrial structure remodeling in a different animal model [10,23–26]. Therefore, previous studies have reported that the use of TZDs was independently associated with a decreased risk of NAF [11,27]. In a study by Chao et al., the relative risk for NAF in individuals taking a TZD after adjustment for age, underlying diseases, and baseline medication compared with that in individuals not taking a TZD was 0.69 (95% CI 0.49–0.91) [11]. However, our result is contrary to that reported by the RECORD [8] and PROactive [9] studies, which enrolled high-risk patients with type 2 DM and did not show a reduction in NAF in the TZD group compared with that in the non-user group. With a mean age of approximately 70 years, the patients in our study had an average 4-year history of diabetes. Moreover, less than 3% had preexisting clinical cardiovascular disease, and the comorbidity rate was low, including a hypertension prevalence rate of 20%. The differences between our findings and those reported by the RECORD [28] and PROactive [29] studies may be attributable to between-study differences in patients’ baseline characteristics. The pathophysiological mechanism for patients with higher cardiovascular risk (as in PROactive and RECORD) deriving no benefit from TZDs in terms of AF prevention, whereas for those with lower risk (as in our study) benefiting from TZDs in terms of having a lower risk of AF remains unknown [30, 31]. Further studies to evaluate the pathophysiological association between NAF risk and TZDs are needed.

Insulin is the most commonly used second-line antihyperglycemic drug for patients with poor glycemic control [32] Furthermore, previous studies have reported that the duration of DM and glycemic control are related to the development of NAF [33–36]. Dublin et al. found that there was an association between hemoglobin A1c (HbA1c) levels and the risk of NAF in a population-based cohort study [33] They indicated that a patient with poor glycemic control with a 1% higher HbA1c level was associated with a 14% (OR 1.14, 95% CI 0.96–1.35) risk of developing NAF. In our study, insulin was found to be associated with a high risk of NAF. A potential explanation for the association between insulin therapy and NAF may be that DM on insulin are those with longer duration of diabetes (S1 Table), poor control of diabetes on oral agents prior to going on to insulin and also having significant other comorbidities.

Sulfonylureas, glinides, α-glucosidase inhibitors, and DPP4 inhibitors were not associated with the risk of NAF in the current study. Several studies have reported that DM is one of the independent risk factors for the development of NAF [23, 37, 38]. The reason for these findings in our study is unclear, but it may be attributable to the innate character of patients with DM. These findings emphasize the need for further investigation of the mechanistic links between these antihyperglycemic drugs and NAF.

Some limitations of this study need to be emphasized. First, all patients in this study were recruited from claims datasets of the Taiwan NHI that had been submitted by primary care clinics. The risk factors for atrial fibrillation such as family history, treatment adherence, severity of DM, baseline HbA1c or glucose levels, and diet were not available from these secondary data. This is an important limitation. However, because the data we used were population-based data, we assumed that there were no differences among the seven antihyperglycemic groups. Second, all cases in this study were collected from claims datasets of primary care clinics, and prescriptions were based on physicians reporting only in Taiwan; therefore, our findings cannot be generalized to patients in different areas. Third, the diagnosis of AF in this study was based on ICD-9 codes. Given that AF may be asymptomatic, and no systematic screening was performed to identify silent AF, there is a high probability that many AF cases were missed. Therefore, the potential for misclassification bias exists. Additionally, no distinction was made between paroxysmal and persistent AF during subject recruitment. Finally, this is a sex-, age-, DM duration-, and Charlson Comorbidity Index score-matched randomly selected case study, and we have adjusted all available confounding factors (including chronic kidney disease, AMI, etc.). The clinical relevance of this study must be further established through large-scale prospective trials.

### Conclusions

In conclusion, our results show that NAF patients were more likely to have used insulin than non-NAF patients and less likely to use biguanides or TZDs. Biguanides and TZDs may be associated with a lower risk of NAF, whereas insulin may be associated with a greater risk of NAF, but those findings require confirmation through a cohort design. Although an association between antihyperglycemic agents and NAF was found, these data do not establish causation. However, these findings may provide important clinical implications for the mechanistic links between antihyperglycemic drugs and NAF in DM patients. Further prospectively randomized studies are needed to confirm the generalizability of the present study.

## Supporting information

S1 Table Diabetic duration of all antihyperglycermic drugs use. The diabetic duration for users of insulin was significant longer than that for non-users (*p* < 0.05). However, the differences of diabetic duration of all antihyperglycermic drugs use are very small (2 to 6 months), which is likely not clinically significant.
(DOCX)
