# Supplementary material for: Antihyperglycemic drugs use and new-onset atrial fibrillation: A population-based nested case control study
Source: PLoS One. 2018 Aug 30;13(8):e0197245. doi: 10.1371/journal.pone.0197245 (PMC6116917; doi:10.1371/journal.pone.0197245)
Supplement: S1 Table — The diabetic duration for users of insulin was significant longer than that for non-users (p < 0.05). However, the differences of diabetic duration of all antihyperglycermic drugs use are very small (2 to 6 months), which is likely not clinically significant. (DOCX) [file pone.0197245.s001.docx]

S1 Table. Diabetic duration of all antihyperglyceric drugs use

| Variable | Non-AF group | AF group | p-value |
| --- | --- | --- | --- |
| Insulin, mean (SD) Y | 3.8 (2.8) | 4.4 (2.8) | <0.001 |
| Biguanide, mean (SD) Y | 4.0 (2.9) | 3.9 (2.8) | 0.229 |
| Sulfonylurea, mean (SD) Y | 3.9 (2.9) | 4.0 (2.8) | 0.001 |
| Glinide, mean (SD) Y | 3.9 (2.9) | 4.2 (2.8) | <0.001 |
| α-glucosidase inhibitor, mean (SD) Y | 3.9 (2.9) | 4.2 (2.8) | <0.001 |
| Thiazolidinedione, mean (SD) Y | 3.8 (2.8) | 4.3 (2.8) | <0.001 |
| DPP-4 inhibitor, mean (SD) Y | 3.9 (2.9) | 4.1 (2.8) | <0.001 |

Mean(SD) Y: Mean + standard deviation years.

DPP: dipeptidyl peptidase.
